# Human Temporal Cortical Single Neuron Activity during Language: A Review

**DOI:** 10.3390/brainsci3020627

**Published:** 2013-04-26

**Authors:** George A. Ojemann

**Affiliations:** Department of Neurological Surgery, University of Washington, Box 356470, Seattle, WA 98195, USA; E-Mail: gojemann@u.washington.edu; Tel.: +1-206-543-3575

**Keywords:** language, human single neuron activity, recent verbal memory, verbal associative learning

## Abstract

Findings from recordings of human temporal cortical single neuron activity during several measures of language, including object naming and word reading are reviewed and related to changes in activity in the same neurons during recent verbal memory and verbal associative learning measures, in studies conducted during awake neurosurgery for the treatment of epilepsy. The proportion of neurons changing activity with language tasks was similar in either hemisphere. Dominant hemisphere activity was characterized by relative inhibition, some of which occurred during overt speech, possibly to block perception of one’s own voice. However, the majority seems to represent a dynamic network becoming active with verbal memory encoding and especially verbal learning, but inhibited during performance of overlearned language tasks. Individual neurons are involved in different networks for different aspects of language, including naming or reading and naming in different languages. The majority of the changes in activity were tonic sustained shifts in firing. Patterned phasic activity for specific language items was very infrequently recorded. Human single neuron recordings provide a unique perspective on the biologic substrate for language, for these findings are in contrast to many of the findings from other techniques for investigating this.

## 1. Introduction

There are multiple methods for investigating the human brain organization for language. Each provides a somewhat different perspective. They fall into two groups: those that identify essential regions, as a function fails when that brain region is inactivated, and those that identify regions that participate in a function but are not necessarily essential for it. Among the former are the effects of lesions, electrical stimulation mapping and intravascular neuroparalytic drugs. The latter group includes the metabolic correlates of a function as measured by functional magnetic resonance imaging (fMRI), and “optical imaging” [1] and the electrophysiologic correlates in the scalp electroencephalogram (EEG), electrocorticogram (ECoG) and microelectrode recordings of single neuron activity and local field potentials (LFP). The relation between these different techniques has been an active area of recent research, for example between techniques identifying essential regions (by electrical stimulation mapping) compared to those regions participating in a function (by fMRI) [2] or between different techniques for identifying participatory regions (fMRI compared to microelectrode recording) [3]. Here we review findings from microelectrode recordings from the author’s laboratory on the changes in single neuron activity during simple language measures, along with findings from recordings during recent verbal memory and verbal learning measures that help explain some of the language findings. 

All human microelectrode studies are conducted in clinical settings. This imposes a number of constraints. All are from special populations, those with the conditions whose treatment provides the clinical setting. In the studies reviewed here, the clinical setting is cortical resections for the treatment of medically refractory epilepsy with a technique where the patient is awake under local anesthesia for a portion of the procedure [4]. This technique is used so that the individual patient’s resection can be tailored to epileptic activity in the ECoG unperturbed by general anesthesia and the location of crucial areas for language based on intraoperative electrical stimulation mapping [5]. Since the surface of cortex has no sensation, and with modern propofol anesthesia the patient can be asleep for placement of the local anesthesia blocks and the craniotomy and then quickly awakened, this technique provides a several hour period where the patient can be awake and participate in cognitive measures for electrical stimulation mapping and the investigative microelectrode studies reported here.

The majority of the patients with the criteria for this procedure have seizures arising in temporal lobe. As a result, this review is further limited to findings in temporal cortex. Although recordings avoid tissue and neuronal activity that shows physiologic changes that have been related to the epileptic process, the extent to which findings can be generalized to other populations, including “normals” is generally not known, as there are no current techniques for obtaining these or similar data in “normals”. Moreover, these studies are limited to that portion of the patient population who consent to participate in them, and, because microelectrode recording is invasive, to tissue that will be subsequently resected to treat that patient’s epilepsy. This last restriction means that there are no recordings from cortex identified as essential for language in that patient, based on electrical stimulation mapping.

The intraoperative extracellular microelectrode recordings reported here include information on the action potential firing from 1 to 4 single neurons, separated by the configuration of the action potentials. It is generally thought that this reflects a random sample of activity of medium to large pyramidal neurons. We have used 1–4 such electrodes in our studies, sampling one or two cortical sites. At rest, these neurons have remarkably low firing rates, not infrequently less than 1 Hz. This is not unique to human temporal cortex for it has also been observed in lateral temporal cortex of awake monkey [6]. Peak activity with various tasks commonly averages around 20 Hz in well isolated neurons with maximum recorded values up to 50+ Hz. The studies reviewed here include data from 9 to 34 patients, yielding 17–105 neurons each. These intraoperative microelectrode recordings thus reflect changes generalized across the patient population rather than many sites in an individual.

Human single neuron recording began with the work of Arthur Ward, Jr., first Professor and Chairman of Neurological Surgery at the University of Washington [7]. The author’s first experiences with this began when he joined Dr. Ward’s program in 1960 as a neurosurgery resident. The initial interest was in characterizing the changes in neuronal activity with epilepsy [8,9]. Reports on human neuronal activity during cognitive measures began appearing in the 1970’s, in the work of Bechtereva *et al.* [10] in recordings from thalamus and inferior frontal lobe, and Halgren *et al.* [11] from medial temporal lobe. The author’s studies reviewed here were initiated in 1985 [12]. Despite the passage of over 25 years since then, most of the reports of changes in human neuronal activity during cognition have come from these centers; in recent years from the UCLA program predominately investigating activity in medial temporal lobe [13] and the author’s program predominately investigating lateral temporal cortex. Each program has used different recording and analytic techniques and different cognitive measures as well as recording in different parts of the brain, so that there has been little attempt by different centers to replicate others’ findings. 

The unusual setting and limited amount of time available for intraoperative microelectrode recordings has a number of effects on the design of the cognitive measures used in these studies. Complex tasks have been avoided. Some measure indicating that the task was performed has been included, for verbal measures an overt indication that items were perceived and verbal motor mechanisms intact. Control measures have been intermixed with task items. In the author’s studies control measures are defined contrasting behaviors rather than “rest”.

## 2. Findings

### 2.1. Language

Most of our investigations of single neuron changes with language have utilized simple tasks. Initial studies [14,15,16] investigated auditory word perception alone and with overt production of the word. Tones at 500 or 1000 Hz, random noises of the environment, speech degraded by removing spectral components and speech backward provided controls for activity related to auditory perception. Additional studies, each in a separate series of patients, investigated object naming, overtly and silently [12,17,18,19]. Comparison was to matching a spatial feature on the same items used for the naming task. Single word identification was studied, in conjunction with naming [12,18,19] or in other patient series as the only identification task [20,21]. The control items in these last studies were the words backwards, while matching of a spatial feature on the word items was the behavioral control for the other studies. The earliest auditory studies were descriptive, while the other studies established changes statistically. 

Despite this variety of language and control tasks and methods of analysis, several features of the single neuron activity changes with the language tasks are evident in all the studies. Changes for all tasks were not lateralized to the language dominant hemisphere but rather present in about equal proportion in either hemisphere. For example, in the two studies that have data from both hemispheres for overt naming and word reading, during naming 30% of the 50 neurons sampled from left hemisphere had significant changes in firing rate, and 32% of the 28 neurons sampled from the right [18,19]. For word reading, 20% of 46 neurons sampled in left hemisphere, 32% of 25 from right. This is in marked contrast to language changes present with lesions or electrical stimulation mapping of temporal cortex, where the changes are confined to the dominant hemisphere. 

**Figure 1:**
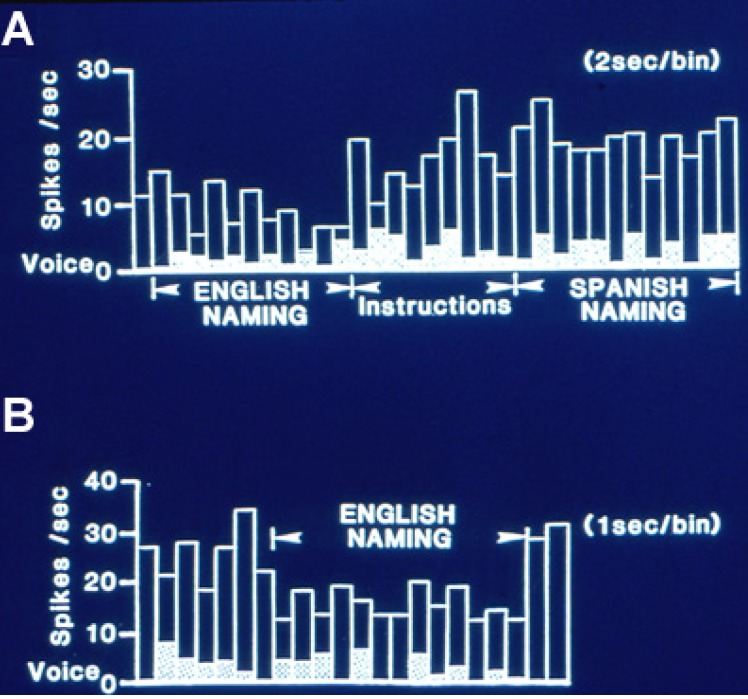
Single neuron activity from left, dominant superior temporal gyrus during overt naming of the same object pictures in two languages, English and Spanish. (**A**) Activity in 2 s bins during English naming. That shifts to a higher level with the instruction to name the same pictures in Spanish. Activity then continued at the same high level for some minutes during English and Spanish reading and the spatial matching tasks to the naming and reading slides. (**B**) Activity, now shown in 1 s bins, then abruptly decreased again during repeat naming in English, returning to the higher level on its completion. This recording illustrates the relative inhibition with naming characteristic of dominant temporal cortical activity, and the differential activity with the same linguistic task in different languages. Adapted from [23].

In the earliest auditory listening and repeating studies activity changes recorded from superior temporal gyrus were predominately increased activity during listening, while that from middle gyrus was more frequent during repeating and predominately decreased activity, a relative inhibition, with a variable onset sometimes preceding voice onset by up to 250 ms [14,15]. This effect was also identified during object naming when it was found during both overt and silent naming, commonly with onset immediately after item presentation. Indeed inhibition represented 75% of the left hemisphere changes in activity with overt naming in the studies of Schwartz *et al.* [18] and Ojemann and Schoenfield-McNeill [19]. Although no lateralization of this relative inhibition was identified in the early study of auditory word listening and repetition, in the later studies of object naming early inhibition was shown to be significantly lateralized to the dominant hemisphere and late excitation to the nondominant hemisphere [18]. Early inhibition was also a feature lateralized to the dominant hemisphere during a rhyming task [22]. Figure 1 is an example of this relative inhibition occurring during naming. This effect was less evident during reading tasks where no feature of those changes could be significantly lateralized.

Several hypotheses for the mechanism of this relative inhibition have been proposed. Initially it was identified during the auditory word repetition task as excitation during perception and inhibition during the overt response. It was suggested as a mechanism to block perception of one’s own voice during overt speech. However, subsequently inhibition was also identified during naming and to a lesser extent, reading, including onset with perception and sustained throughout the task (Figure 1) even with silent tasks, indicating that blocking perception of one’s own voice is not the full explanation. The inhibition may represent an inhibitory surround, perhaps around an area of increased activity at a site crucial for language based on stimulation mapping, from which we have not recorded. That inhibition is less evident with word reading may support this concept, as stimulation mapping suggests that anterior temporal cortex may be somewhat more likely to be crucial for reading than naming [24] and thus possibly have more excitatory activity. Alternatively, the inhibited neurons may be part of the network for the language task that is active with learning of new aspects of the task, but inhibited during the overlearned aspects such as those tested with object naming or word reading. We will return to this hypothesis later in this paper when discussing the changes in activity with verbal associative learning. Finally, cortical activity may modulate subcortical circuits, such that reduced cortical activity allows the higher subcortical firing rates that have been recorded during language [25]. Early inhibition may be a more general property of functional lateralization in temporal cortex, for a similar effect was lateralized to nondominant hemisphere recordings during a visuospatial task, face matching [26].

A third feature present in multiple series of recordings during language measures is the frequent separation of activity for different aspects of language. Neurons changing activity in the same direction with naming and reading have been infrequently recorded, representing only 9% of the 63 neurons recorded during both tasks in the studies of Schwartz *et al.* [18] and Ojemann and Schoenfoeld-McNeill [19]. Separation of neurons changing activity with naming in one language and not another have been identified in the few recordings obtained in bilinguals (Figure 1). These findings suggest that the networks that subserve different aspects of language are at least partially separate, a findings similar to that for crucial cortical areas for different aspects of language identified by stimulation mapping [24,27,28]. Nor are naming and reading networks represented in nearby neurons. Extracellular microelectrode recordings can sometimes be separated into activity from nearby neurons based on action potential configuration. In the 21 sets of 2 or 3 nearby neurons recorded in the Schwartz *et al.* [18] and Ojemann and Schoenfield-McNeill [19] studies, none had changes for naming and/or reading in more than one neuron of the set. 

In monkey, neurons in frontal cortex have been identified that have similar changes in activity with perception of a movement and its production [29]. It has been suggested that these “mirror” neurons had a role in the development of language, a concept reinforced by the finding in monkey of frontal lobe neurons “mirroring” sounds of a movement and that movement’s production [30]. Stimulation mapping identified frequent overlap in human superior temporal gyrus of sites where changes in the orofacial movements of speech production and the perception of phonemes were evoked [31]. However, single neuron recording from temporal cortex during measures of language perception and production have much less clearly demonstrated this overlap, more often having significant changes with perception or production alone [14,15,18,19,32], or as indicated above, when present for both the combination of excitation for perception and inhibition for production. In the studies of Schwartz *et al.* [18] and Ojemann and Schoenfield-McNeill [19], 70% of the neurons assessed during naming that had changes in perception or production or both had changes in only one. The proportion was similar for reading (67%) and did not differ between hemispheres. A minority of neurons in human temporal cortex that change activity during language measures have changes that could represent “mirror” properties. Similar to findings with naming compared to reading changes, close proximity of neurons with perceptual or production changes also does not seem to be the case. The stimulation mapping findings then presumably reflect the presence of separate neurons in the networks for either function in the larger volume of tissue affected by stimulation. 

**Figure 2:**
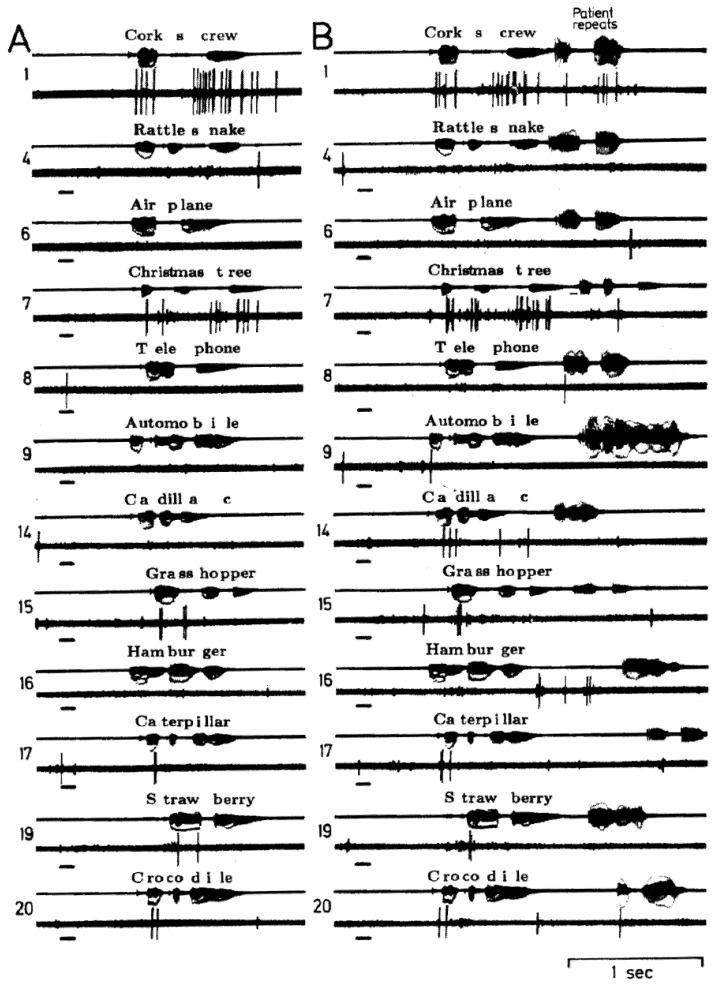
Single neuron activity recorded from right superior temporal gyrus during an auditory language task, listening (**A**) and listening and repeating (**B**) the same 20 words. The short bar to the left in each sample is a 1000 Hz tone. The patient’s overt correct repeating of the word during B is the activity on the far right of the audio channel, the remainder reflects the open field presentation of the words from an audio tape. This neuron appears to have the same patterns of activity during the two sample of listening to words 1, 7, 15, 17, 19 and 20 and an absence of activity during both samples of listening to the other words. This pattern was thought to reflect responses to specific phoneme categories. There is little to no activity in response to the tone or with overt repeating. Adapted from [14].

**Figure 3:**
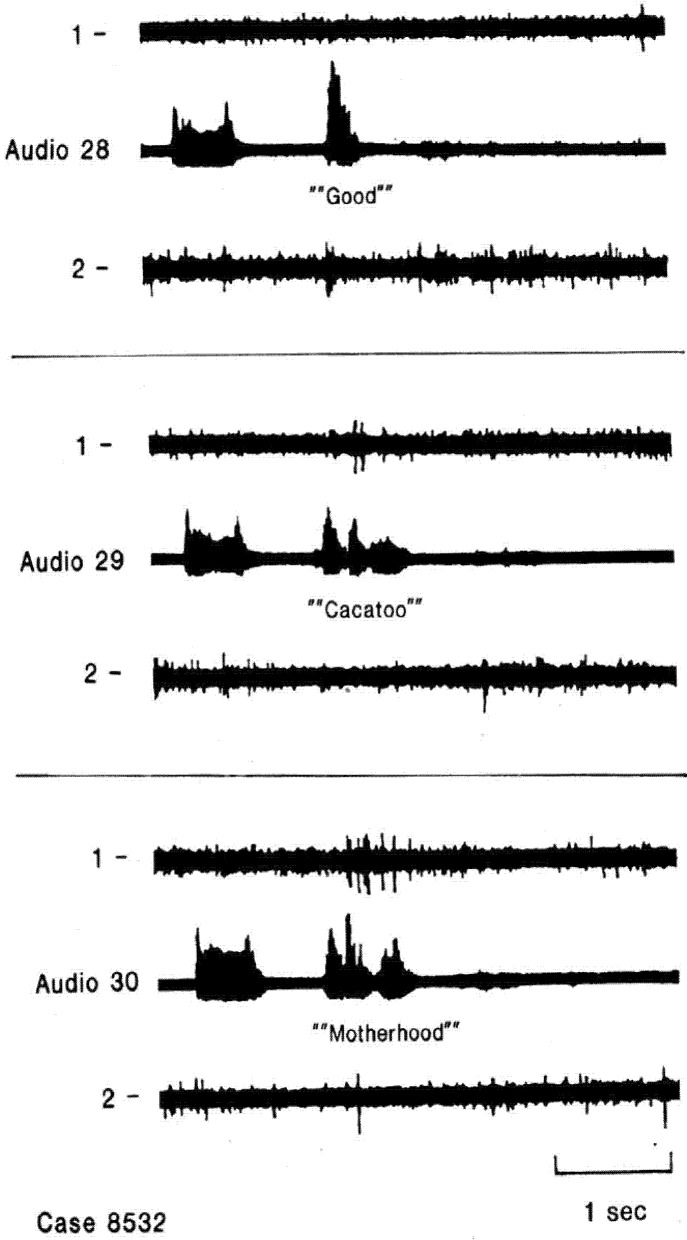
Single neuron activity related to second and subsequent syllables. Recordings from two electrodes at different depths in the same region of nondominant superior temporal gyrus during word listening. Neuron in channel 1 responds only to second and subsequent syllables of multisyllable words and not to single syllable words. Creutzfeldt and Ojemann, unpublished data [36].

The changes described above represent alterations in firing rates. However, a widely hypothesized model of the neuronal activity related to language involves temporal patterning. Some evidence for such temporal patterning related to language has been suggested based on recordings from subcortical nuclei [33] and a mechanism for temporal patterning of activity of individual neurons involving interactions with local field oscillations in the “gamma” range has been proposed [34]. In our lateral temporal recordings during language measures, we have identified only a few examples that seem to show some element of temporal patterning. During the auditory tasks, a neuron was identified during auditory listening that seemed to have the same patterns of activity during perception of particular phonemes ([14], Figure 2). Another neuron (in another patient) had similar selectivity to both auditory listening and overt repeating of the same word [15]. A few other neurons seemed to have patterns related to word structure, during auditory listening responding selectively to the second but not first syllable of multisyllable words (Figure 3) and also only compound words [14]. During the recent verbal memory paradigm described below, another neuron in another patient had significant overrepresentation of the interspike intervals in the 10–25 msec bins at encoding and retrieval, the two tasks requiring perception or production of the same name, compared to the different names used as distractors during memory storage [35]. These are hints, then that there may be temporal patterns for different words, perhaps reflecting different features of the word: phonemic structure, prosody, perhaps semantics, but if present, temporal patterns seem to be very sparsely coded. This question, of the role of temporal patterning compared to firing rate changes remains an important area for future investigation.

### 2.2. Relation between Language and Verbal Memory

The activity of lateral temporal cortical neurons has also been assessed during a measure of recent verbal memory [12,17,19,20,21,37]. Each trial of that measure had encoding, storage and retrieval phases, where encoding was naming of objects, reading of visually presented words, or auditory presentation of words, information that was then retained over a storage phase filled with distractors of similar modality, followed by cued overt recall. Because of the time constraints on intraoperative microelectrode recording, the storage interval was commonly limited to nine seconds. The type of memory assessed by this measure has often been called “working” memory, but differing from some working memory paradigms by the presence of distractors during the storage phase, but it also involves encoding, storage and retrieval of specific explicit semantically identified information, and thus would seem to also assess explicit verbal memory.

In our studies, activity during the encoding phase of the memory measure was compared to that during identification of the same material, but without the requirement of retaining that information in memory. With this comparison any perceptual, motor and semantic memory components should be subtracted as the subject’s task during the two conditions is identical, overtly identify verbally the object or word so that the two conditions differ only by the instruction to retain the material in memory. But the result is a major difference in the activity of these same neurons. In contrast to the changes with overt identification described in the previous section, many more neurons are now active. When the 239 neurons recorded from 86 subjects from the publications cited above are pooled, the presence of the instruction to retain the material in memory changed activity during encoding in 57% with the majority of this activity excitation. There is now no lateralized difference in inhibition, though the late excitation lateralized to the nondominant hemisphere during the overt portion of the tasks remains [38]. Some of the neurons inhibited during the language tasks are activated during memory encoding. Separation of the regions of temporal cortex crucial for verbal identification or the encoding phase of a similar memory paradigm has been identified with electrical stimulation mapping [39,40].

The nature and timing of this encoding change has been examined in detail [38]. A change in activity sustained throughout encoding was the most common pattern, lateralized to dominant hemisphere, with sustained inhibition a characteristic of middle temporal gyrus activity. Activity confined to perception and early processing occurred earliest in superior temporal gyrus, most often excitation. Indeed some of that excitation peaked remarkably early, within 50–150 ms of item presentation. Activity associated with overt identification of the item to be remembered was lateralized to the nondominant hemisphere, most often increased activity in the 300 ms preceding initiation of the overt response. A convergence of sustained and early excitation was observed in 10% of the neurons sampled. In about half of the neurons with sustained encoding changes these extend into the storage phase of the memory measure. That activity likely represents an active rehearsal process [41]. A minority of neurons have significant changes in the comparison between the encoding, storage and recall stages, with those neurons significantly more likely in inferior temporal gyrus and basal temporal recordings compared to superior and middle temporal gyrus recordings [37]. 

Additional insight into the relation between language and verbal memory came from recordings from neurons with activity that discriminated correct from incorrect overt identification of verbal material [42]. Neurons with that property were over represented in medial basal and hippocampal recordings, significantly less frequently present in lateral middle temporal gyrus recordings with none found in superior temporal gyrus. Since medial temporal structures have usually been related to recent explicit memory and not language, including the semantic memory component of language, this finding was unexpected, but may indicate that explicit memory for specific items is a component of language in addition to that general semantic memory, so that the error related activity may be a part of an ongoing monitoring of responses to the specific items. Although the study included identification of auditory, text and named objects, and neurons were recorded that discriminated errors in each modality, no neuron had activity that discriminated more than one modality, further evidence of the separation of networks for different language modalities. In 69% of the neurons discriminating correct from incorrect performance, there was a relative inhibition for correct identification that was absent with errors, evidence of the importance for this inhibition in language. Activity discriminating correct from incorrect identification was present earlier in lateral temporal cortical neurons (the majority within 300ms of item presentation) than in medial basal neurons. 

### 2.3. Relation between Language and Verbal Learning

A substantial literature has established the importance of the temporal lobe in learning. We have assessed this at a neuronal level in lateral temporal cortex, in two separate series of patients [20,21]. Both studies used a verbal associative learning paradigm where a set of words, common concrete nouns, were visually presented under three conditions: with the instruction to read the word aloud, in the recent memory paradigm described above, and in a paired-associate learning task, where the words were presented as unrelated pairs in a series of encoding-test trials. During the encoding portion the subject read aloud each pair. During the test portion, the first word of each pair was presented. The subject read this aloud and then provided the associated word, if known. The encoding-test trials were repeated up to 7 times, with pairs in different orders between trials and encoding and test portions of each trial. Words backwards were the perceptual control. In the first study activity during silent reading of the words was also assessed. The first study recorded activity from 49 neurons in 15 patients, the second 21 neurons from 9 patients. Twenty-eight of the 70 neurons of the combined studies were from right hemisphere.

In both studies, the majority of neurons had significant changes in activity with associative learning, 59% and 62% respectively. That proportion was similar between the hemispheres. Increased activity accounted for 71% of those changes. Neurons were also classified by the change in activity during identification and recent memory. Combining the two studies, 47% of the neurons were related to identification, 66% to recent memory. Changes were equally divided between excitation and inhibition for identification, with 66% of the memory changes excitation. In both studies, the neurons with changes during word identification and memory were significantly more likely to have associative learning changes than neurons unrelated to either. In the initial study, neurons with both identification and memory changes had significantly greater activity for associations learned early compared to those learned later or never. Once learned that activity rapidly decreased, within 2 further correct trials. The pool of increased neuronal activity shrinks once learning occurs, an effect that has also been observed with functional imaging [43] and stimulation mapping [44]. Subjects who learned the associations poorly had less activity in these neurons than subjects who learned the associations rapidly and conversely, the poor learners had greater activity in neurons with no relation to identification or memory, substantiating the functional significance of the increased activity in these neurons. 

The second study compared activity for associations learned on the first encoding trial to that for the remaining unlearned associations. Neurons with significantly *decreased* activity with identification and increased activity with memory encoding had relatively greater activity for associations learned on that first encoding trial than those not. Neurons with other relations to identification or memory did not show this effect. In that study the first encoding trial was subdivided by the temporal epochs related to pair presentation and the overt verbal identification of the pair Both learned and unlearned pairs showed an increase in activity from the period immediately before pair presentation, to 300 ms before overt identification, but activity for learned pairs was at a significantly higher level during overt identification sustained into the interval before presentation of the next pair. 

In summary, learning a new association to a verbal item involves a sustained increase in activity in those neurons that are inhibited with item identification. We hypothesized that those neurons were active with initial learning of that verbal item, but once learned, rather rapidly inhibited as part of the general reduction in activity once learning occurs. These neurons are reactivated with the learning of new associations to the verbal item. That reactivation represents sustained “tonic” activity lasting beyond perception and identification of the new association, not unlike the sustained activity related to rehearsal we identified during recent memory storage. It is this dynamic change in activity in the existing network for a verbal item that seems to characterize associative learning, rather than recruitment of new neurons into the network, at least in temporal cortex. Moreover, the associative learning changes are similar to those occurring with recent memory encoding, though more widespread.

### 2.4. Local Networks Involved in the Relation between Language and Associative Learning in Temporal Cortex

Our recent investigation of the ability of local field potentials (LFP) to predict spike timing in lateral temporal neurons provides additional insights into the organization of the neural networks related to language and associative learning [45]. The data for that study were derived from recordings during a word reading and paired associate learning paradigm designed to be suitable for both intraoperative neuronal recording and functional magnetic resonance imaging (fMRI), in an effort to identify any neuronal correlates of fMRI changes in human temporal association cortex that occurred at the same temporal lobe sites, in the same subjects, during the same behavioral task [3]. This paradigm involved alternate blocks of trials of associate learning or identification of words, performed silently and each with a relatively large number of rapidly presented (1/s) items, but otherwise similar to the previous learning paradigm. Testing of performance after each block of associative learning demonstrated significant learning. As part of this study not only was single neuron firing assessed, but also LFPs recorded through the same electrode. In the study of Zanos *et al.* [45], those LFP signals in different frequency bands from 4 to 150 Hz were convoluted with a Volterra kernel, and the timing of action potentials predicted by the output of the kernel was compared to the actual discharges. The relation of spike activity to LFP oscillations was also assessed. This study also included a number of control measures to insure that the LFP signal was not contaminated by any signal from action potentials. 

In the 69 single neurons from 14 patients included in this study, significant prediction of spike timing (to within 1 ms) was present for 25 neurons in spike-free recordings, with from 7% to 49% of spikes correctly predicted. These significant cases formed two groups. For 14 “high frequency” neurons, spike timing was significantly more frequently predicted by high LFP frequencies (80–150 Hz) than low (4–14 Hz). In 10 other “low frequency” neurons, it was the reverse. These two groups did not differ in power spectra of their LFPs, spike waveform, or pattern of firing. They differed in the contribution of different components of the kernel, in the relation to LFP oscillations and to the behavioral measures. “High frequency” neurons had large contributions from second order kernel, low from first order. Low frequency had more phase locking to LFP oscillations (4–10 Hz). Mean firing rate for the “high frequency” neurons was significantly higher than that of “low frequency” neurons for the paired-associate learning blocks, while “low frequency” neurons had higher firing rates than “high frequency” neurons during the identification blocks (although as in previous studies firing rates of all neurons were higher during associative learning than identification).

The importance of these findings to an analysis of the networks related to identification and learning is based on the analysis of these signals in experimental animals. The LFP is considered to reflect the postsynaptic inputs to a small volume of tissue around the electrode, within 250 microns diameter. The low frequency component is thought to represent activity projected from distant neuronal populations, the high frequency from local neuronal circuits. The activity with associative verbal learning then represents that of local neurons, the activity with identification effects projected from greater distances. Projecting this onto our model of the changes between relative inhibition (reduced firing rate) with identification transiting to excitation with learning of a new association to that item, the inhibition is a distant effect, perhaps favoring an inhibitory surround, the excitation a new local effect which, given its “tonic” nature might be triggered by mechanisms such as a “thalamocortical” activating system.

## 3. Conclusions

Human temporal cortical single neuron recordings during language measures have provided a number of unique insights. The proportions of neurons changing activity with language tasks (and verbal memory and learning) are very similar between hemispheres. This is in marked contrast to the left lateralization of language effects of lesions or electrical stimulation mapping. In temporal lobe, the networks for different language tasks such as naming and reading or naming in two languages are largely separate. Indeed, more than one network is rarely represented in very nearby neurons recorded through the same microelectrode. This separation seems to extend to activity related to language perception or production, although networks for both are present in either temporal lobe. 

There is a surprising amount of relative inhibition, reductions in activity with language tasks. This is particularly prominent during naming, when it is significantly lateralized to the dominant hemisphere. Some of this anterior temporal “inhibition” seems to occur during overt speech, where it may act to block feedback of one’s own voice. But the majority seems to represent a dynamic portion of the network, where these neurons become at least briefly active with encoding of new verbal material into recent memory, and even more so with learning of new verbal associations to the same items, suggesting that they may have been active with initial learning of those items, and then actively inhibited as those items became overlearned. There is evidence that the reduction in activity reflects input from relatively distant portions of the network, in contrast to the increases with memory and learning that seem to reflect local inputs. Most of this activity represents tonic sustained shifts in firing. Neurons with these properties represent a surprisingly large proportion of the sample of lateral temporal neurons from which we have recorded, often over half. Although it is often assumed that there is patterned phasic activity for specific language items, such activity has so far been infrequently recorded, suggesting that such neurons are sparsely represented in anterior temporal cortex.
